# Polymeric Hydrogel Coating for Modulating the Shape of Keratin Fiber

**DOI:** 10.3389/fchem.2019.00749

**Published:** 2019-11-20

**Authors:** Lanlan Wang, Artur Cavaco-Paulo, Bo Xu, Madalena Martins

**Affiliations:** ^1^International Joint Research Laboratory for Textile and Fiber Bioprocesses, Jiangnan University, Wuxi, China; ^2^Centre of Biological Engineering (CEB), University of Minho, Braga, Portugal; ^3^Key Laboratory of Eco-Textiles, Ministry of Education, Jiangnan University, Wuxi, China

**Keywords:** hydrogel, redox polymerization, coating, keratin fiber, morphological modification

## Abstract

Hydrogel coating was explored to modulate the shape of keratin hair fiber. The motivation was the development of an eco-friendly methodology with non-toxic chemicals to modulate keratin fiber. Polymeric hydrogel of acrylic acid and N-N-dimethylacrylamide was prepared by free-radical polymerization in aqueous solution, using nano-alumina particles as crosslinker and potassium persulfate as an initiator. Physico-chemical properties of the hydrogel was investigated by Fourier transformer infrared spectrum (FTIR), thermal analysis and swelling ratio behavior. After hydrogel coating, morphological modification was observed from straight to curly hair effect. The influence of hydrogel coating on hair fiber was evaluated by perming efficiency supported by X-ray diffraction and morphological characterization (SEM and AFM). The durability of hydrogel coating was tested until four wash processes maintaining around 65% the new configuration of the hair fiber.

## Introduction

Hydrogels are polymer networks which are swollen material with water and possess three-dimensional structure with tuneable physicochemical properties. The polymeric matrix is formed by crosslinking polymers chains produced by the reaction of one or more monomers using cross-linking agents. Polymeric hydrogels have been described as water-swollen network possessing high flexibility and good biocompatibility (Ahmed, 2015; Vundavalli et al., 2015; Nesrinne and Djamel, 2017). Due to their unique properties, hydrogels have received considerable attention in several applications including tissue engineering (Lee et al., 2011), wound healing (Kamoun et al., 2017), superabsorbents (Guilherme et al., 2015), and drug delivery systems (Li and Mooney, 2016). Hydrogels are also suitable bioadhesive for biomedical and cosmetic applications (Caló and Khutoryanskiy, 2015; Parente et al., 2015; Liu et al., 2018).

Hair fiber has a highly organized and cylindrical structure, formed by keratinized cells following a defined design. Owning to it arrangement, forms a rigid structure in the molecular level leading to mechanical and flexibility resistance (Velasco et al., 2009). Hair fiber is hygroscopic, and absorbs a large amount of water moisture which binds by hydrogen bonds to the aminoacids (Zviak, 1986). The social importance of healthy hair and the continuous desire to manipulate the hair morphology have been the driving force for significant research in the last decades. The specific characteristics of each type of hair and the treatments applied affect in overall visual appearance. Successful cosmetic treatments to change the shape of the hair require chemical processes (Harrison and Sinclair, 2003; Sinclair, 2007). These chemical processes have the reduction phase based on reductive agents such as thioglycolic acid, on lye-based relaxer such as sodium hydroxide, on non-lye-relaxer such as calcium hydroxide, or guanidine hydroxide, among others. During the reduction phase disulphide bonds are broken and newly cysteine bonds were formed. After that, followed the reduction phase based on oxidizing agents (hydrogen peroxide), where the hair assumes the new configuration rebuilding the disulphide bonds (Johnson, 1997). These harsh chemical agents are toxic and carcinogenic (Tyl et al., 2003), and could lead to severe damages in hair keratin fiber, damages in neck, scalp and hands as well as release toxic elements for the environment (Kaur et al., 2002; Mcmichael, 2007; Maneli et al., 2014; Miranda-Vilela et al., 2014; Galli et al., 2015). As a result of the damage, hair changes it physico-mechanical properties leading to reduced cross-linking density and can suffer from the appearance of crack and lifting of the cuticle (Robbins, 2002; Dyer et al., 2013). From here, can occur the removal of the hair cuticle hydrophobic top-layer, and consequent reduction of surface hydrophobicity (Cruz et al., 2017). The development of processes with non-toxic chemicals to manipulate the shape of the hair (Xu et al., 2017) and maintaining it integrity still remains an open challenge.

In a previous study, hydrogel-coated keratin fiber determined dimensional changes of wool yarns under humidity conditions (Wang et al., 2018). Hydrogels can be prepared by free radical polymerization crosslinking one or more monomers in the presence of an initiator and a crosslinking agent (Sarac, 1999; Martens et al., 2002; Ahmed et al., 2016). Redox initiation has been widely applied to initiate polymerization reactions which has industrial importance including low-temperature emulsion polymerization. Redox polymerization is an effective method in the generation of free radicals under mild conditions to initiate the reactions (Sarac, 1999; Zhang S. et al., 2016). In this study, a hydrogel solution precursor was synthesized to investigate it coating influence on the manipulation of the shape of Asian hair fiber. Here, the hydrogel coating was applied, as illustrated in Graphical Abstract onto hair fiber surface by immersion into the hydrogel solution. The polymerization of acrylic acid and dimethyl acrylamide in aqueous solution with potassium persulfate as an initiator in the presence of a metal based crosslinking agent was developed. Experiments were performed to assess the physico-chemical and mechanical properties of the hydrogel coatings. To evaluate the effect of hydrogel coatings on hair keratin fiber different procedures were conducted and morphological characteristics of the fiber was analyzed through changes in terms of curls and length of the hair. The durability of the hydrogel coating to maintain the new configuration effect of the hair was assessed through fourth washing processes. The most of polymeric materials are known to be non-toxic/non-irritable in contact with skin (Fiume, 2002; Pemberton and Lohmann, 2014).

**Graphical Abstract:**
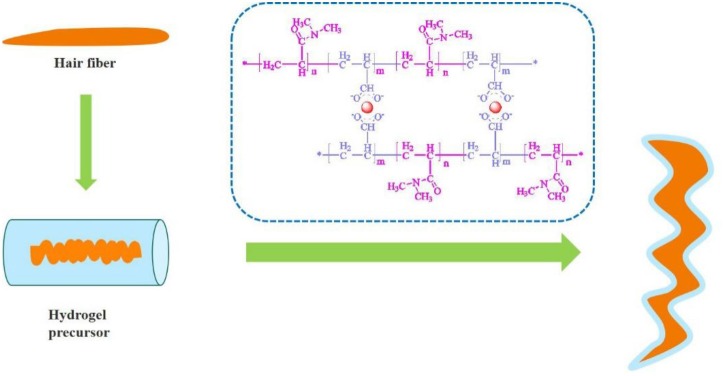
Modulating the shape of keratin fiber by polymeric hydrogel coating.

## Materials and Methods

### Materials

Hair samples were provided by a barbershop originated from Asia (Wuxi- China). Urea, sodium hydroxide and potassium persulfate (KPS) were obtained from Sinopharm Chemical Reagent Co., Ltd. (Shanghai, China). Acrylic acid (AA) was purchased from Aladdin Industrial Corporation, Shanghai, China. N, N-dimethylacrylamide (DMAA) was supplied from TCI, Shanghai, China. The nano-alumina nanoparticles (Al_2_O_3_ NPs) dispersion with size ranges from 5 to 10 nm was obtained from Hangzhou Wanjing New Materials, China. All the other reagents were used as received.

### Preparation and Coating of the Hydrogel on Hair Fiber

**Figure 1:**
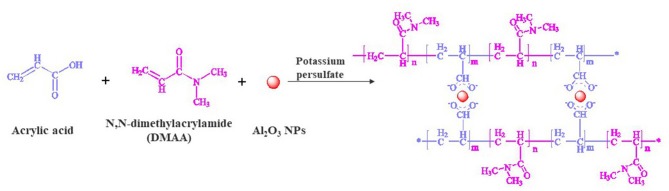
Hydrogel formation with nano-alumina oxide particles in the presence of potassium persulfate.

The hair tresses were washed with a commercial shampoo and dried at room temperature. Each hair tress was weighted for around 1 gr. Before hydrogel coating, the hair tresses were subjected to a pre-treatment with and without urea followed by the coating with the hydrogel. The polymeric hydrogel precursor was based on nano-alumina oxide nanoparticles as crosslinking agent, Acrylic Acid (AA) and N, N-dimethylacrylamide (DMAA) as monomers, and Potassium Persulfate (KPS) a powerful oxidant used as initiator of the polymerization. Figure 1 represents the reaction mechanism for the hydrogel formation. The initiator solution was produced as follow: 0.2 gr of KPS was dissolved in 10 mL de-ionized water, for 30 min with stirring under nitrogen atmosphere.

The reaction solution with monomers and crosslinking agents was stirred for 30 min under nitrogen atmosphere. Then, was added the initiator (0.5 mL from the previous solution) in the reaction solution at 0–4°C. The polymerization was carried out in an ice-water bath for 20 min with stirring.

The coating procedure for human hair samples were performed under two sequences. The Asian hair samples (step 1) were wrapped in the glass rod (step 2) and immersed in water or immersed in a pre-treatment solution of urea 8M (step 3). Then, the hair was removed from the glass rod (step 4) and freely wrapped (step 5) was immersed in the hydrogel precursor solution (sequence 1). For sequence 2, the hair is maintained in the glass rod (step 4) followed immersion in the hydrogel precursor solution (step 5).

The coating of the hydrogel on the hair fiber was performed for 12 h at 0–4°C. After that, the hair tresses were washed thoroughly with commercial shampoo and dried at room temperature.

### Fourier Transform Infrared (FTIR) Spectroscopy

The chemical structure of the synthesized polymeric hydrogel was analyzed by using Fourier Transform Infrared spectroscopy (FTIR). Dried sample of the hydrogel was used. The FTIR spectrum was recorded over the range of 500–4,000 cm^−1^, using the KBr pellet method on a Fourier transform infrared spectrophotometer (Nicolet iS 10, Thermo Fisher Scientific, USA).

### Differential Scanning Calorimetry (DSC) and Thermogravimetric Analysis (TGA)

The thermal properties of hair samples were examined using a DSC equipment Q200 V24.8 Build 120 and a TGA equipment Q500 V20.13 Build 39 from TA Equipment Company of the United States. The temperature range covered was around 25–400°C for DSC and 40–800°C for TGA analysis. The scanning rate was 10°C/min under dynamic nitrogen atmosphere and the weight of measured samples was 5 ± 0.5 mg.

### Measurements of the Swelling Ratio

The swelling ratio experiments were conducted weighting the dried and the humid hydrogel. To that, a dried hydrogel sample cut into sheets with thickness of 2 mm and diameter of 6.3 mm, and then 10 sheets were weighed. Then, the samples sheets were immersed in deionized water at room temperature and after that the hydrogel samples sheets were removed from the water and weighted. Swelling measurements were conducted in triplicate for each sample. The swelling ratio (SR) was calculated using the equation:

(1) $$\begin{array}{r} \text{SR}=\frac{Ws-Wi}{Wi}\times 100\% \end{array}$$

Where *Ws* is the weight of the swollen sample sheets, *Wi* is the initial (dried) weight of the hydrogel sample sheets. The swelling ratio is expressed as percent weight ratio.

### Qualitative and Quantitative Evaluation of Hair Treatments

Before all treatments the hair samples were washed with a commercial shampoo thoroughly to clean it. Hair tresses were evaluated qualitatively and quantitatively, from visual effect to calculating perm efficiency (PE) for each treatment, respectively. Statistical analysis of the experimental results was performed using analysis of variance (ANOVA). Differences were considered statistically significant at *p* < 0.05. The data were represented as mean ± standard deviation of three measurements.

Hair chemical treatment, a commercial product, included two steps, the reduction and neutralizing. In the first step, the straight hair was wrapped around rods to generate curls, and a basic solution of ammonium thioglycolate was applied for 20 min. The reduced hair was rinsed with water and dried with a towel. The second step involves the oxidation reaction using a neutralizer agent (hydrogen peroxide). Finally, the hair was rinsed thoroughly with water.

Perming efficiency (PE) was calculated using Equation (1).

(2) $$\begin{array}{l} PE=\\ \frac{n^{\underline{\text{o}}}loops\;after\;treatment/fiber\;lenght\;after\;treatment\;}{n^{\underline{\text{o}}}loops\;before\;treatment/fiber\;lenght\;before\;treatment}\;\times 100 \end{array}$$

### Wide-Angle X-Ray Diffraction

X-ray diffraction studies were conducted on a diffractometer model D2 PHASER (Bruker AXS Co. Germany). Nickel-filtered Cu (Kα) radiation, with a wavelength of 0.154 nm, generated at 30 kV and 10 mA was used. Diffraction patterns were obtained by continuous scanning at angular region of 2θ range from 5 to 90°.

### Tensile Properties of Hydrogel Coated Human Hair

The uniaxial tensile tests were characterized on the MIT-1 universal test machine (Sanfeng Co., China) with a 50 N load cell. The hair samples treated with different conditions were measured at room temperature. The data obtained were each calculated from an average of ten single hair yarns. Percentage of elongation at break was calculated by the elongation at the moment of rupture divided by the initial length measurement and then, multiplied by 100.

### Characterization of Hair Surface Morphology-SEM and AFM

The morphological characterization of hair was examined by scanning electron microscopy (SEM) technique in an electron microscope SU1510 (Hitachi Co. Ltd., Tokyo, Japan), at an acceleration voltage of 20 KV at 500x of magnification.

Atomic force microscopy (AFM) studies were performed using an atomic force microscopy model Dimension ICON, Brook Technology Co, Ltd., Germany. Imaging was obtained in the contact mode fixing the hair specimens on a glass piece.

## Results and Discussion

The hydrogel network preparation by redox polymerization in aqueous media has been broadly used in advanced materials for industrial applications from agriculture to consumer care products (Sarac, 1999; Omidian and Zohuriaan-Mehr, 2002; Tomar et al., 2007; Braun, 2009; Zhang W. et al., 2016). In this method, the solution of monomers in the presence of a crosslinking agent and an initiator are required for the polymerization system occur. Our previous study established a polymeric hydrogel to coat wool yarns, since its microstructure is quite similar to the hair keratin fiber herein we used the same components (Wang et al., 2016, 2018). Two monomers with nano-alumina nanoparticles as crosslinking agent were used once favored the strength of hydrogel coating on keratin fibers. Here, the polymeric hydrogel was synthesized by a reaction solution of acrylic acid and N, N-dimethylacrylamide using nano-alunina nanoparticles as crosslinker, in the presence of potassium persulfate (initiator). The polymer chains were produced by vinyl monomer radicals which undergo propagation to form network hydrogel. The nano-alumina nanoparticles act as crosslinker with acrylic acid monomer, and the potassium persulfate triggered the polymerization reaction. Nano-alumina nanoparticles were used due to the covalent bond onto polymer crosslinked network based on acrylic (Herranz et al., 2012). Metal or metal oxide nanoparticles as nano-alumina particles have great potential in polymer materials because of its unique characteristics and tunable properties, such as responsiveness to magnetic, electrical stimuli and/or to light (Biondi et al., 2015; Zhao et al., 2015). In fact, the incorporation of such nanoparticles into hydrogel network by a covalent manner leads to a relatively strong interaction between polymer and nanoparticles allowing to a remarkable mechanical properties and swelling behavior of hydrogel (Zhao et al., 2015).

**Figure 2:**
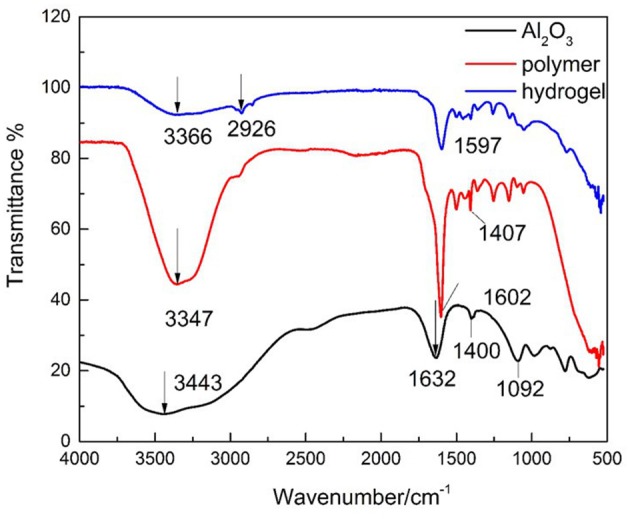
FTIR spectra of (black line) nano-alumina nanoparticles, (red line) polymer (AA-DMAA), and (blue line) hydrogel.

Characterization by FTIR was investigated revealing that polymer 3D network was successfully formed (Figure 2).

Figure 2 display the FT-IR spectra of alumina nanoparticles, neat copolymer of AA with DMAA, and dried hydrogel. FTIR analysis reveals the non-appearance of vinyl bands at 1,680 cm^−1^ indicating that polymerization reaction of all monomeric groups occurred (Ahmed et al., 2016). From FT-IR spectra of neat copolymer (AA-DMAA) the broad absorption peak at 3,347 cm^−1^ is ascribed to the existence of –OH and –NH groups (Singhal et al., 2009) and the band at 1,602 cm^−1^ can be attributed to carbonyl groups of acrylamide and carboxylic acid (Sadeghi and Heidari, 2011; Bhattacharyya and Ray, 2014). Compared to the spectra of hydrogel network, the characteristic band of –OH (3,366 and 1,597 cm^−1^) was deeply weakened which indicates the absence of –OH groups in the hydrogel network (Sarac, 1999), and a strong interaction between carboxyl groups and alumina nanoparticles. According to the literature, the carboxylate group (-COO^−^) act as bridging ligand with the aluminum atoms on the surface of alumina nanoparticles (Barron, 2014) which contributed to the crosslinking network of hydrogel. These results indicate that the incorporation of alumina nanoparticles as crosslinked agent contributed to the formation of hydrogel.

**Figure 3:**
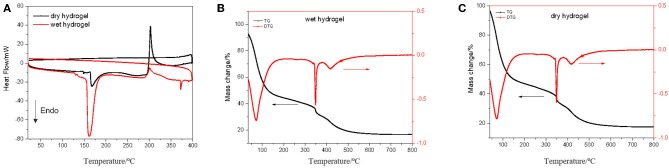
TGA **(A)**, DSC **(B)**, and **(C)** thermograms of hydrogel.

Thermal analysis of the hydrogel samples, DSC and TGA (Figure 3), was an important parameter to characterize the polymeric hydrogel behavior on dry and wet state. TGA analysis was carried out by scanning in the temperature range of 50–800°C at a rate of 10°C/min under a nitrogen atmosphere and flow rate 10 mL/min. From TGA thermograms were observed similar curves for wet and dry hydrogel.

**Figure 4:**
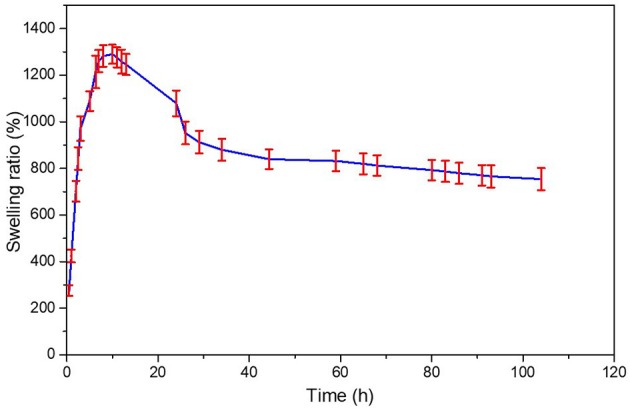
Swelling percentage ratio of hydrogel network in deionized water (*n* = 3, mean ± standard deviation).

It was observed a first event between 50 and 110°C which is related with water loss by about 40%, the range from 110 to 340°C is the second event related with mass loss of about 15% and the third event 340–450°C related with 15% mass loss. From here it is possible to verify that the hydrogel remains reasonable stable in a large scale of temperature. DSC curve of dry hydrogel exhibited one sharp endothermic peak at about 160°C which can be attributed to the loss of water, and a very slight exothermic peak at around 300°C. For dry hydrogel showed a very slight endothermic peak at 160°C and an exothermic peak at 300°C related with release of energy by mass loss. The endothermic peak of individual monomers, 86.38 and 130.38°C for acrylamide ad acrylic acid, respectively (Shah, 2000), did not appear in the synthesized hydrogel, which indicates that a new polymeric hydrogel has been formed. Dynamic swelling study was conducted by measuring the dried and humid weight of the hydrogel immersed in deionized water. The swelling profile of the hydrogel is given in Figure 4.

Measurements of the initial and swollen weight of the hydrogel at time intervals allowed analyzed the swelling percentage ratio of the hydrogel until it reached swelling equilibrium. From Figure 4 it is observed a fast swelling rate of the hydrogel in the initial 10 h meaning that the water was absorbed into the hydrogel increasing the weight of the hydrogel with time. Then, the swelling rate decreased until reached an equilibrium after 40 h. Due to it swelling ratio the hydrogel application on hair fiber could has the ability to prevent a hydrophilic profile of the fiber conserving the moisture in hair. Additionally, also could has the ability to maintain the moisture in hair fiber and eventually reduce hair electrostatics and sustaining the hair style.

**Figure 5:**
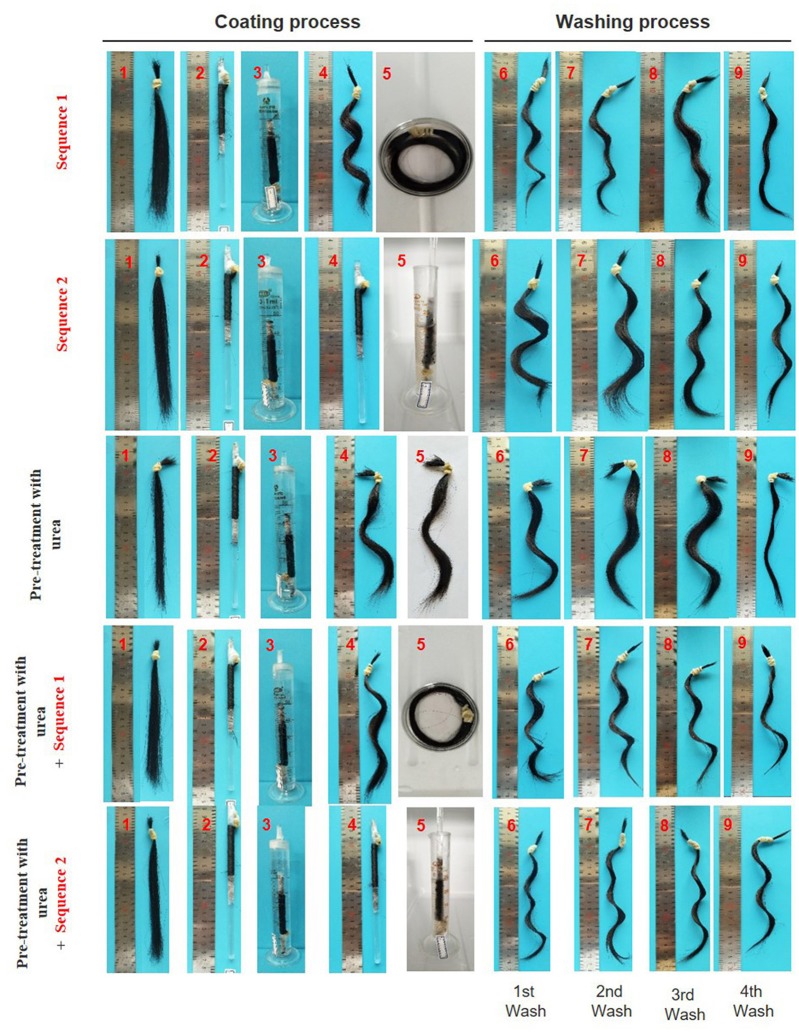
Images of hair samples during/after coating and washing procedure; sequence 1: the hair was removed from the glass rod (step 4) and freely wrapped was immersed in the hydrogel precursor solution (step 5); sequence 2: the hair wrapped in the glass rod was immersed in the hydrogel precursor solution (step 5); pre-treatment with urea (8M); pre-treatment with urea followed by sequence 1; pre-treatment with urea followed by sequence 2.

The coating procedure for human hair samples were performed under two sequences. The hair samples were immersed into the hydrogel precursor solution wrapped off the glass rod and wrapped in the glass rod, sequence 1 and 2, respectively. As shown in Figure 5, the immersion into the hydrogel precursor solution (step 5) it is predominant in the formation of a firm “S” curl.

**Figure 6:**
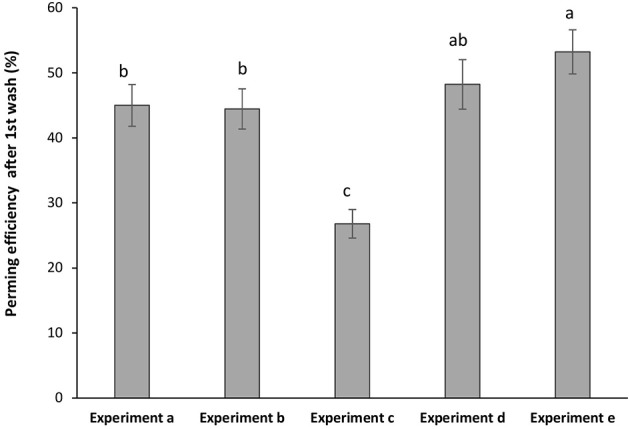
Perming efficiency of hydrogel-coated hair keratin fiber with experiments (a) hydrogel-coated hair by sequence 1; (b) hydrogel-coated hair by sequence 2; (c) pre-treatment with urea (no hydrogel coating); (d) hair pre-treated with urea and coated by sequence 1; and (e) hair pre-treated with urea and coated by sequence 2. Grouping information using Tuckey test with 95% of confidence meaning that do not share a letter are significant different.

The curly effect of the Asian hair was deeply pronounced when keratin hair fiber was coated by the polymeric hydrogel (Figure 6). Samples of pre-treated hair followed by the hydrogel coating obtained higher perming efficiency after first washing. Highest perming efficiency was observed for (experiment e). The film deposited onto the swollen hair fiber allowed a synergistic effect between keratin fiber and hydrogel indorsed changes in hair modulation. The resistance to the washing process was analyzed for all samples. It was found that after the fourth washing process the samples maintained around 65% of the perming effect. For sample without hydrogel coating (experiment c) only maintained 30% of the initial perming efficiency which itself was very low.

**Figure 7:**
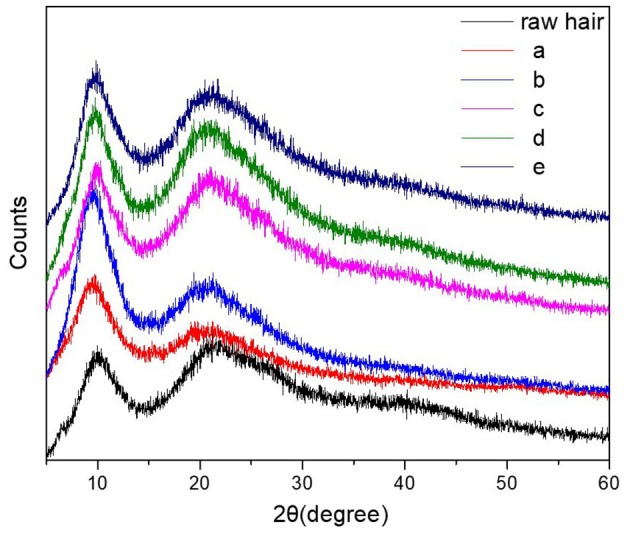
XRD profiles of the raw hair fiber and keratin hair fiber coated by hydrogel network: (a) hydrogel coating (sequence 1); (b) hydrogel coating (sequence 2); (c) pre-treatment with urea (8M); (d) hydrogel coating (sequence 1) with pre-treatment; and (e) hydrogel coating (sequence 2) with pre-treatment. Sequence 1 coating off the glass the rod and sequence 2 coating in the glass rod.

XRD and mechanical analysis allowed to evaluate the effect of the hydrogel coating on crystallization and mechanical properties of the hydrogel coated keratin hair fiber. X-ray diffraction is an effective analysis to determine crystallization. Figure 7 presents the XRD profiles of hydrogel-coated keratin hair fiber [five different procedures as described in detail above; (a) and (b) hydrogel coating sequence 1 and 2, respectively (c) treatment only with urea; (d) and (e) pre-treatment with urea followed by hydrogel coating sequence 1 and 2, respectively] and raw hair fiber. It can be observed that hair keratin fiber, coated and raw hair fiber, presented common diffraction peaks at 10° and 20°, which are assigned to the α-helix and β-sheet structures (Ma et al., 2013; Shen et al., 2015). The raw hair fiber showed that the peak at 10° was broad and the peak at 21° was weak, while the peak at 21° was strengthened for hydrogel-coated keratin hair fiber with a pre-treatment with urea. This indicates that the β-sheet conformation was efficiently preserved by keratin hair fiber pre-treated with urea and coated by hydrogel network. The change of the amount of β-sheets after coating demonstrated structure changes in the hair fiber.

Since the X-ray analysis demonstrated that the keratin hair fiber structure is changed by the hydrogel coating pre-treated with polar solvent (urea), so analyse it influence on mechanical properties it is of high interest. Tensile tests tabulated in Table 1 shows that tensile strength and elastic modulus of the hair fiber was favored by pre-treatment with urea followed by hydrogel coating. The hair fiber become more flexible and less brittle by adding the urea treatment, and this effect was more pronounced when the hydrogel coating in the glass rod was performed. Polar solvents such as urea promotes higher swelling behavior. Since they disrupt inter- and intra-molecular hydrogen bonds and weak hydrophobic interactions between polypeptide chains of keratin (Du et al., 2015; Shen et al., 2016). Once swelling of hair fiber is favored (Velasco et al., 2009; Gavazzoni Dias, 2015), a better penetration of organic molecules occurred, and due to the synergistic effect between keratin fiber and hydrogel, the hair fiber behave less rigidly.

**Table 1:** Tensile properties of hairs: elongation, tensile strength, and elastic modulus for different treatments were calculated.

**Experiments**	**Elongation at break/%**	**Tensile strength/kPa**	**Elastic modulus/kPa**
Experiment a	51.5 ± 7.4	3.3 ± 1.6	48.9 ± 5.4
Experiment b	48,0 ± 8.1	3.8 ± 2.1	71.9 ± 4.4
Experiment c	44.3 ± 6.4	2.9 ± 1.6	51.8 ± 3.8
Experiment d	48.1 ± 7.3	3.2 ± 1.8	45.2 ± 6.1
Experiment e	43.4 ± 7.5	3.0 ± 1.4	41.2 ± 5.8

*Five samples were tested for each experiment*.

**Figure 8:**
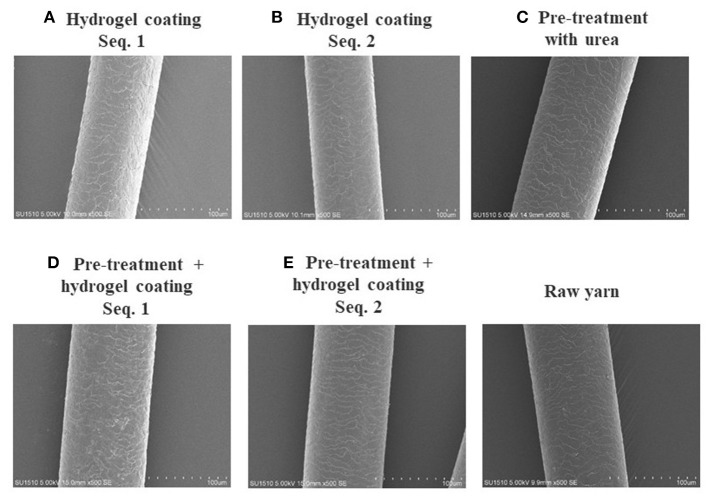
Scanning electron microscope images of hair coated with polymeric hydrogel precursor: **(A)** hydrogel coating (sequence 1) without pre-treatment; **(B)** hydrogel coating (sequence 2) without pre-treatment; **(C)** pre-treatment with urea (8M); **(D)** hydrogel coating (sequence 1) with pre-treatment; and **(E)** hydrogel coating (sequence 2) with pre-treatment. Sequence 1 hair coating off the glass rod and sequence 2 hair coating in the glass rod.

**Figure 9:**
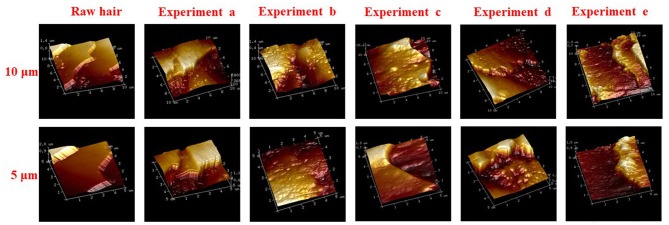
AFM images of raw and treated keratin hair fiber deposited on glass substrate, experiments (a) hydrogel-coated hair by sequence 1; (b) hydrogel-coated hair by sequence 2; (c) pre-treatment with urea (no hydrogel coating); (d) hair pre-treated with urea and coated by sequence 1; and (e) hair pre-treated with urea and coated by sequence 2.

The hydrogel was internalized on keratin fiber surface, and morphological changes of hair fiber coated and uncoated, was observed by SEM and AFM. The surface of the hair cuticle in raw yarn and in hair treatments based on hydrogel coating (Figure 8), revealed a regular overlapping cuticles and a closely adherent pattern. However, coated hair keratin fibers also presented a slight deposition due to the hydrogel film on hair surface. AFM three-dimensional images (Figure 9) presented detailed coated hydrogel hair surface of 10 and 5 μm thick sections onto glass slides. The polymeric hydrogel exhibited rough surface and their amount of material dispersed on the surface. Hair surface roughness indicates the adsorption of the hydrogel on the hair fibers suggesting the interaction between hair surface and hydrogel.

## Conclusions

Morphological modification of keratin hair fiber was exhibited using hydrogel coating methodology. Hydrogel coating was effective in the formation of a new hair configuration. Perming efficiency calculation and visual appearance supported by the X-ray diffraction analysis demonstrated changes in the hair fiber after coating. It was found that changes in the hair fiber showed a film deposition on hair surface which increased surface roughness suggesting an interaction between keratin fiber and hydrogel. The good swelling ratio of the hydrogel could allow the maintenance of the moisture in hair fiber which can reduce hair electrostatics and sustains the hair style. Parameters like treatment time needs to be adjusted to realistic period for application on humans. These findings could lead to new cosmetic applications, where monomers and polymerization initiator could be bottle separately.

## Data Availability Statement

The raw data supporting the conclusions of this manuscript will be made available by the authors, without undue reservation, to any qualified researcher.

## Author Contributions

LW: experimental execution. AC-P: experimental design. BX: co-orientation. MM: co-orientation.

### Conflict of Interest

The authors declare that the research was conducted in the absence of any commercial or financial relationships that could be construed as a potential conflict of interest.
